# Biallelic *MED29* variants cause pontocerebellar hypoplasia with cataracts

**DOI:** 10.1038/s41431-025-01918-6

**Published:** 2025-07-31

**Authors:** Leo Arkush, Geeske M. van Woerden, Limor Ziv, Dina Marek-Yagel, Reginald Fonseca, Esmee Brevé, Ortal Barel, Nechama Shalva, Alvit Veber, Yair Anikster, Dominique Ben-Ami Raichman, Banan Musallam, Shai Marcu, Andreea Nissenkorn, Hanna Mandel, Steven A. Kushner, Bruria Ben Zeev, Gali Heimer

**Affiliations:** 1 Pediatric Neurology Unit, The Edmond and Lily Safra Children’s Hospital, Sheba Medical Center, Ramat Gan, Israel; 2 Dept Neuroscience, Dept of Clinical Genetics and Erasmus MC Center of Expertise for Neurodevelopmental Disorders (ENCORE), Erasmus MC, Rotterdam, The Netherlands; 3 Cancer Research Center, Sheba Medical Center, Ramat Gan, Israel; 4 Metabolic Disease Unit, The Edmond and Lily Safra Children’s Hospital, Sheba Medical Center, Ramat Gan, Israel; 5 Dept Neuroscience, Erasmus MC, Rotterdam, The Netherlands; 6 Genomic Unit, Sheba Cancer Research Center, Sheba Medical Center, Ramat Gan, Israel; 7 Gray Faculty of Medical and Health Sciences, Tel Aviv University, Tel Aviv, Israel; 8 Department of Diagnostic Imaging, Sheba Medical Center, Ramat-Gan, Israel; 9 Pediatric Neurology Unit, Emek Medical Center, Afula, Israel; 10 Department of Pediatrics, Baruch Padeh Medical Center, Poriya, Israel; 11 Pediatric Neurology Unit, Wolfson Medical Center, Holon, Israel; 12 Unit of Inherited Metabolic Disorders, Ziv Medical Center, Safed, Israel; 13 Institute of Human Genetics, Ziv Medical Center, Safed, Israel; 14 Department of Psychiatry, Columbia University Irving Medical Center, New York, NY USA; 15 SNF Center for Precision Psychiatry & Mental Health, Columbia University, New York, NY USA

**Keywords:** Genetics research, Experimental models of disease

## Abstract

Pontocerebellar hypoplasia (PCH) represents a group of disorders characterized by cerebellum and pons hypoplasia, variable cerebral involvement, microcephaly, severe global developmental delay (GDD), and seizures. We sought the genetic cause of PCH in two siblings. Genetic workup was performed by whole-exome sequencing followed by Sanger validation. Morpholino-knockdown zebrafish embryos with human wild-type gene rescue were used to assess cerebellar development and motor function. Transfected mouse hippocampal cultures and electroporated mouse embryos were employed to assess functional effects on neuronal morphology and development. Both patients presented with profound GDD, severe microcephaly, cataracts, and variably seizures. Their MRIs demonstrated marked cerebellar and pontine hypoplasia. Both were homozygous for a c.416T > C, p.(Leu139Pro) *MED29* variant which was predicted to be pathogenic. Locomotion and cerebellar GABAergic neurons development were both impaired in *MED29* Morpholino-knockdown zebrafish and rescued by human wild-type gene expression. ShRNA-knockdown of *MED29* in mouse hippocampal neurons decreased neurite length and arborization in vitro, and caused defective embryonic neuronal migration in vivo. Overexpression of MED29 p.(Leu139Pro) was consistent with a loss-of-function. Taken together, the Mediator complex regulates transcription processes, and defects in particular subunits are associated with distinct neurodevelopmental phenotypes involving PCH. We conclude that *MED29* is a novel risk gene for PCH.

## Introduction

Pontocerebellar hypoplasia (PCH) describes a group of neurodegenerative disorders, with significant clinical and neuroradiological variability, typically characterized by limited acquisition of developmental milestones, motor impairments, severe intellectual disability, and epilepsy, associated with dysfunction of the cortex, cerebellum, and basal ganglia. An increasing number of genetic defects associated with distinct subtypes have been identified. The most prevalent is PCH2A, which is related to biallelic pathogenic variants in *TSEN54* (OMIM 277470) involved in transfer-RNA splicing [1].

Mediator (MED) is a large multiprotein complex with a role in RNA polymerase II (Poll II) gene transcription. Although most of the MED subunits have not been shown to be associated with human disease, there are accumulating descriptions of severe neurodevelopmental disorders arising from pathogenic variants in specific subunits of the complex. Homozygous variants in the genes coding for *MED11* (OMIM 620327), *MED17* (OMIM 613668), *MED20* (OMIM 612915) and *MED27* (OMIM 619286), located in the head region of the complex, were associated with global developmental delay (GDD), microcephaly, spasticity, dystonia, epilepsy and variably cataracts, accompanied by cerebellar and in part pontine atrophy [2–6]. Similar overlapping phenotypes have been described in subunits thought to be in the tail module (*MED23*) (OMIM 605042), as well as in *MED25* (OMIM 610197), whose location within the Mediator complex is poorly defined, with additional cardiac abnormalities [7, 8], suggesting both common and distinct roles for individual subunits within the complex.

Here we present a novel homozygous variant in *MED29* (OMIM 612914), which has been previously unrelated to human disease, associated with severe GDD, microcephaly, pontocerebellar hypoplasia, cataracts and variably epilepsy, including functional and structural modeling in Morpholino-knockdown (Mok) zebrafish, transfected mouse primary hippocampal cultures and knockdown mouse embryos.

## Materials and methods

### Clinical and genetic workup

The proband underwent a thorough neurological examination and workup including brain magnetic resonance imaging (MRI), electroencephalogram (EEG), and metabolic investigations. DNA was extracted from peripheral blood. Trio whole-exome sequencing (WES) of the proband and his parents, followed by bioinformatics, was performed at the Center for Human Genome Variation, Duke University School of Medicine, Durham, North Carolina, USA [9, 10]. Candidate variants were obtained and filtered according to the following exclusion criteria: noncoding and synonymous variants, variants with MAF > 0.01 in healthy controls databases or which were found in a homozygous or hemizygous mode in healthy controls databases, cases where reads supporting variant/total reads <0.25 and variants who were predicted by PolyPhen-2 to be of low pathogenicity. Targeted Sanger sequencing was applied for validation and familial segregation. Whole exome sequencing was later performed on the proband’s younger affected brother at the Sheba Medical Center, Israel.

### Zebrafish strains and maintenance

Zebrafish experiments were conducted at the Sheba Medical Center zebrafish facility. All experiments were performed on zebrafish (*Danio rerio*) between 1 and 8 days post fertilization (dpf) obtained from a laboratory stock of wild-type and transgenic adults. The zebrafish line used in this study was Tg(*huC:Gal4)* [11, 12] along with corresponding wild-type strains. Embryos were raised in egg water supplemented 0.003% N-Phenylthiourea (PTU, Sigma P7629), starting 24 h post-fertilization (hpf) to inhibit melanogenesis. Larvae were raised at 28 °C on a 14 h:10 h light:dark cycle with daily medium changes.

### Morpholino injections

Morpholinos (MOs) were obtained from GENE Tools LLC (Philomath, Oregon, United States). The following translation-blocking MOs (ATG) were used: *Med29MO*^*atg*^ (TGCTCATCTGCTG GGACGCCATTGC), *Med17MO*^*atg*^ (GACCCCCCGACATCATCACCACCTA) and *p53MO*^*atg*^ (GCGCCA TTGCTTTGCAAGAATTG). MOs were injected in concentrations of 0.125 mM (*Med17MO*^*atg*^), 0.2 mM (*Med29MO*^*atg*^), and 0.2 mM (*p53MO*^*atg*^). Approximately 2 nl were injected into the cytoplasm of one-cell-stage zygotes of wild type or tg(*huC:Gal4)* zebrafish using micromanipulator and PV830 Pneumatic Pico Pump (World Precision Instruments, Sarasota, FL, USA).

### Rescue constructs and injections

For rescue experiments, human wild-type (wt) *MED29* 5’ UTR + CDC (3100 bp) was cloned upstream of a 3’ PolyA (238pb) and downstream of a UAS promoter (3127pb) (Gateway®, Invitrogen, ThermoFisher scientific LTD). For rescue experiments, the linearized *hMed29*^*wt*^ construct (5 or 10 ng/μl) was co-injected with 0.2 mM *Med29MO*^*atg*^ and 0.2 mM *p53MO*^*atg*^ into Tg(*huC:Gal4*) embryos. Total RNA was extracted from larvae at age 3dpf and 6dpf for RT-PCR analysis. At 6dpf, larvae were tested for behavioral ‘touch responses’ and then fixed for whole mount ISH analysis. The expression of human *Med29* mRNA in injected embryos was verified by PCR.

### RNA probes and whole-mount in situ hybridization

PCR amplicons of zebrafish *Med17* (NM_001077574.1, L- CGTCAGCATCGAGTCGTC, R- CGCAGGTTATTACGCACAGA and L-GGAGCAGAGAAGCTCTCCAA, R-AGGTCACTGGGAATCTGCAC), *Med29* (NM_173271.1, L- TCCCAGCAGATGAGCACTAA, R- TTTGTCAAACCGCTGAACTG and L-TGTGATCAGCTGGAGCTCTG, R-ACCTTTCCCAGCGATCTTTT), *Pvalb7* (NM_205574.2, L- CATGAAAAACCTTTTGAAAGATGAC R-CGTTTCATCTCATCCTCTTCATGT), *Rora2* (NM_001110167.1, L- CCCCTTACCCCAACTTATGG, R- CCGATCCGAATAACTCCTTG) and *Vglut1* (NM_001098755.1, L- AGGAGAAGGAGCTTCCCATC, R- CAGCCAGCACAAGAAAAGAA) were cleaned from gel, cloned into pGEM-T easy (Promega) and sequenced (ABI PRISM 3100 Genetic Analyzer, AME Bioscience). Clones were used as templates to prepare digoxygenin (DIG)-labeled antisense riboprobes (DIG RNA labeling kit, Roche). RNA probes larger than 500 bp were fragmentized before hybridization to 400–500 bp fragments using alkaline buffer. Whole-mount in-situ-hybridization (ISH) was conducted on embryos treated with 0.003% phenylthiocarbamide as previously described [13]. After whole-mount ISH, larvae were placed in 70% glycerol and imaged under a dissecting stereoscope with a DP72 digital camera (Olympus). According to the intensity and cellular distribution of mRNA expression, embryos were scored as “high” or “low” expression levels in the cerebellum.

### PCR and RTPCR

Total RNA was extracted (TRIZOL) from a pool of 5–10 uninjected and injected zebrafish larvae according to the manufacturer’s protocol. After DNase treatment, total RNA was used as template to prepare cDNA (High-Capacity, Applied Biosystems™) using PolydT primers. cDNA was used as template for RT-PCR to determine the expression levels of the following cerebellar genes: *s100b (gene id* 436825), *Klf9* (565869), *Med29* (797134), *Klf8* (562805), *VglutI*/slc17a7a (795293), *Aldocb (*369193), *Zic1* (30096), *Reelin (*260303), *pValb7* (402807) [14–16]. For expression levels analysis, CTs were compared first to CyclophilinA (ΔCT) in the tissue (*ppiab*, NM_001328424.1) and then to control (ΔΔCT).

### Behavioral test and imaging

At 6 dpf, tg(HUC:GAL4) larvae were injected with *Med29MO*^*atg*^ alone or together with the *UAS:* human*Med29* construct, and scored individually for presence or absence of trunk movement in response to light touch with a fine hairbrush (‘touch response’) [17]. Representative movies of intact and impaired larvae locomotion are provided in the Supplemental Material (Supplementary Videos 1 and 2, respectively).

### Statistical analysis for zebrafish experiments

*Med29MO*^*atg*^ and rescued larvae were analyzed for “touch response” and *Pvalb7* expression in cerebellum (by ISH) in four treatment groups, 3–6 replicates/treatment, *n* = 17–73 larvae/treatment. Differences in the number of fish with high motility or *Pvalb7* expression between treatment groups were tested using one-way ANOVAs followed by t-tests, comparing high-dose versus no or low-dose rescue (JMP®, SAS Institute Inc.). Expression levels in real-time PCR were compared using one-way ANOVAs followed by t-tests.

### Mouse strains and maintenance

Mouse experiments were conducted at the Department of Clinical Genetics at the Erasmus Medical Center. For the primary hippocampal neurons, *FvB/NHanHsd* females (ordered at 6–8 weeks old from Envigo) were crossed with *FvB/NHanHsd* females. For the *in-utero* electroporation experiments, *FvB/NHanHsd* females were crossed with *C57Bl6/J* males (ordered at 6–8 weeks old from Charles River). All mice were group-housed in IVC cages (Sealsage 1145T, Tecniplast) with bedding material (Lignocel BK 8/15 from Rettenmayer) on a 12/12 h light/dark cycle in 21 °C (±1 °C) and humidity at 40–70. Food [801727CRM(P) from Special Dietary Service] and water were available *ad libitum*.

### Primary hippocampal cultures

Adult pregnant female mice were sacrificed via cervical dislocation at E16.5 of gestation. Pups were immediately removed from the uterus, and brain tissue was collected in ice-cold Neural Basal Medium (NB; Gibco, 21103049). Hippocampi were isolated from embryonal brains and collected in 10 mL ice cold NB, after which the tissue was washed twice with cold NB and incubated with Trypsin/EDTA (Sigma; T3924) at 37 °C. The tissue was then washed twice with warm NB and kept in supplemented NB (1%penicillin/streptomycin (Sigma; p4333)/1%GlutaMax (Gibco; 350500-38)/2%B27-supplement (Gibco; 17504044) for dissociation. Single cell dissociated neurons were seeded into 12-well plates containing Poly-D-Lysine (Sigma; P0899) coated coverslips with 1 mL supplemented NB per well. Cells were incubated at 37 °C/5% CO_2_.

### Cloning of the plasmids for neuronal transfections

*MED29*^*WT*^ sequence (Genbank: NM_001321571.2) was obtained from the human brain cDNA library and immediately tagged with restriction sites AscI and PacI by PCR, allowing the ligation of the *MED29*^*WT*^ sequence into TOPO and dual promoter expression vectors, as previously described [18, 19]. Primers used: Fw, 5′ – gaatccggcgcgccaccatgctgaaaagcaacggggag – 3′; Rev, 5′ – ggattcttaattaatcacagagtgcccccagg – 3′. For overexpression, the p.(Leu139Pro) variant was introduced in the *MED29*^*WT*^ sequence in TOPO, using site-directed PCR mutagenesis. Primers used for mutagenesis: Fw, 5′ – accagctggagccgtgcctgcgcct – 3′; Rev, 5′ – aggcgcaggcacggctccagctggt – 3′. Once successfully mutated, the sequence was ligated into the multiple cloning site (MCS) of the expression vector. The same expression vector, lacking an insert in the MCS, was used as a negative control throughout the experiments and referred to as “empty vector control.” For knockdown experiments, three different shRNAs targeting the coding sequence of mouse *Med29* were obtained from the MISSION shRNA library for mouse genomes of Sigma Life Sciences and The RNAi Consortium (TRC) [1]: CCGGTGATTCAGAACACTAACATTGCTCGAGCAATGTTAGTGTTCTGAATCATTTTTG [2]; CCGGGCAGCGCTTTGACAAGTGTTTCTCGAGAAACACTTGTCAAAGCGCTGCTTTTTG [3]; CCGGAGTACCTGGCTGTCATCAAAGCTCGAGCTTTGATGACAGCCAGGTACTTTTTTG. The control shRNA plasmid is the MISSION non-target shRNA control vector: CAACAAGATGAAGAGCACCAA.

### Neuronal transfections and immunological staining

Primary hippocampal neurons (e16.5) were transfected on days in vitro (DIV) 3 with the following constructs for the overexpression experiments: empty vector control, MED29^WT^, or MED29^Leu139Pro^. 2.5 μg DNA was transfected of each construct, except for the empty vector control, in which 1.8 μg DNA was used. For knockdown, a pool of the 3 shRNAs (1ug each) was co-transfected with an RFP plasmid (Addgene). For control, the control shRNA (1 μg) was co-transfected with the RFP plasmid. Neurons were transfected by mixing the DNA with NB and Lipofectamine 2000 according to the manufacturer’s instructions (Invitrogen; 11668-019). Neurons were fixed 5 days post-transfection (DIV8) using 4% PFA/4% sucrose. Fixed cells were labeled for MAP2 (1:500, Synaptic System; #188004) by overnight incubation at 4 °C and visualized with a conjugated secondary antibody donkey-anti-guinea pig Alexa647 (1:200; Jackson ImmunoResearch #706-605-148), incubated for 1 h at room temperature, and finally mounted with Mowiol to allow for fluorescence imaging using confocal microscopy.

### In utero electroporation

*In utero* electroporation was performed as previously described [18, 19]. Briefly, adult pregnant female mice (FvB/NHanHsd) underwent surgery at E14.5 of gestation. After exposing the uterus, embryonic pups were intraventricularly injected with a mixture of DNA construct (1.5–3.0 µg/µl) and FastGreen (0.05%), using the Picospritzer^®^ III, after which a small electrical current was applied (five electrical square pulses of 45 V; duration 50 ms/pulse; duration pulse interval 150 ms; driven by a pulse generator ECM 830, BTX Harvard Apparatus), orientating the tweezer-type pedestal with its positive pool on top of the developing somatosensory cortex. The following plasmids were electroporated for overexpression: Empty vector control; MED29^WT^ or MED29^Leu139Pro^. For knockdown experiments, co-transfection was performed of the pool of *Med29* shRNAs or the control shRNA with an RFP plasmid (Addgene). After the procedure, the mice were returned to their home cage until spontaneous delivery five days post-surgery.

### Perfusion and immunohistochemistry

Pups were sacrificed on the day of birth or one day after birth (P0 or P1; postnatal day 0 or 1, respectively), through cardiac perfusion with saline solution, followed by 4% PFA. After perfusion, the brains were isolated and post-fixed in 4% PFA for 1 h at room temperature. The brains were then stored overnight in 30% sucrose in 0.1 M PB. Perfused brains were embedded in 14% gelatin/30% sucrose, and free-floating sections were made using a cryo-microtome (40–50 µm thick). Sections were washed in 0.1 M PB and counterstained with 4′,6-diamidino-2-phenylindole solution (DAPI, 1:10000, Invitrogen) before being mounted on glass and covered with Mowiol (Sigma). Only a selection of sections was used for counterstaining. Stained and covered brain slices were used for confocal imaging.

### Confocal microscopy

Images were acquired using a LSM700 confocal microscope (Zeiss). For primary hippocampal neurons, images were taken from 8 to 10 transfected neurons per condition (20× objective, 0.5 zoom, 1024 × 1024) per batch of neurons, with a minimum of 2 batches of neurons per condition. For the migration analysis, images were taken from two to three non-consecutive sections from at least three successfully targeted animals per condition (10× objective, 0.5 zoom, 1024 × 1024). Images were analyzed using NIH ImageJ.

### Statistical analysis for mice experiments

#### Neuronal morphology

Total neurite length and number of branches were traced using ImageJ and its plug-in module NeuronJ. Within each batch, the data was normalized to the MED29^WT^, making it possible to pool the normalized data from different batches and allowing application of a one-way ANOVA (Tukey’s multiple comparison test) using Prism GraphPad. For the knockdown, an unpaired two-tailed *t*-test was used. Number of neurons/batches analyzed per condition: empty vector 20/2; MED29^WT^ 17/2; MED29^Leu139Pro^ 19/2; control shRNA 20/2; *Med29* shRNA 20/2.

#### Neuronal migration

Data were assumed to be normally distributed. Statistical analysis was performed on data of the first four bins of each cropped image, corresponding to the cortical plate (CP). The CP was anatomically defined as the most proximal 40% of the dorsoventral distance between the pia and ventricle. In the overexpression experiments, all conditions were compared to empty vector control and to MED29^WT^ overexpression. A one-way ANOVA followed by Tukey’s post-hoc test for multiple comparisons was performed for the overexpression experiment. For the knockdown an unpaired, two-tailed t-test was used. Cumulative graphs were made as a visual indication of the general migration pattern but were not used for statistical analysis. For all conditions, images of at least 3 separate pups (2–4 images per pup) were used for the migration analysis. Number of images used per condition: empty vector 14; MED29^WT^ 9; MED29^Leu139Pro^ 8; control shRNA 15; *Med29* shRNA 15. PRISM software (Graphpad 8.0) was used for the statistical tests. *P* values < 0.05 were considered statistically significant.

## Results

### Case studies

**Fig. 1:**
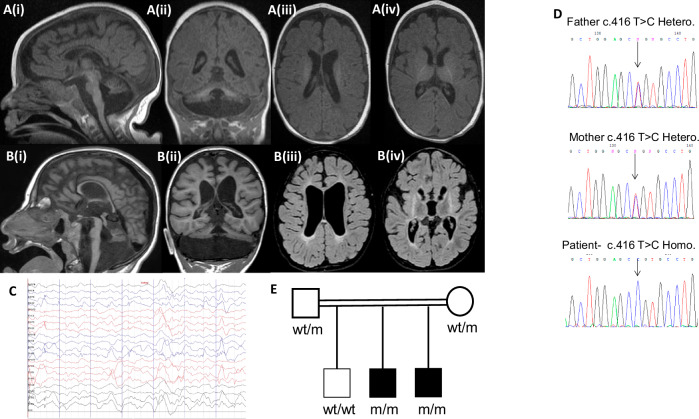
Clinical data of the proband and his family. **A** Brain MRI of proband performed at aged 5 months (T1-weighted image: i – sagittal, ii – coronal, iii/iv – axial) demonstrating prominent hypoplasia of all cerebellar structures, hypoplastic pons, generalized mild cerebral atrophy, thin corpus callosum, and delayed myelination. **B** Brain MRI of proband’s brother aged 4 years, 5 months. (T1-weighted image: i – sagittal, ii – coronal) demonstrating profound hypoplastic cerebellum including the vermis and the pons with a thin corpus callosum. Axial T2/FLAIR- weighted image (iii/iv) shows brain atrophy, diffuse diminished white matter, very small basal ganglia with high T2/FLAIR signal signifying damage/gliosis, with preserved thalamus structure. **C** Inter-ictal EEG recording from the proband demonstrating dysrhythmia and multifocal epileptiform activity. **D** Chromatograms of the proband and his parents. Top – the heterozygous parents, bottom – the homozygous proband. **E** Pedigree of affected family. Solid black squares indicate affected individuals, wt – wild type, m - c.416T > C *MED29* variant.

The proband was born to healthy, consanguineous parents of Middle Eastern Arab descent. Pregnancy and birth were unremarkable. He was born at full term with a birth weight of 2.5 kg, head circumference (HC) of 32 cm (1.71 standard deviations [SD] below the mean) and an Apgar score of 9 at minutes 1 and 5. Bilateral cataracts were noted prior to discharge, for which he later underwent surgery. He presented to our tertiary center for neurological evaluation at the age of 10 months with profound GDD, severe microcephaly (HC 40 cm, −4.71 *SD*), an exaggerated startle response, and myoclonic seizures. Brain MRI performed aged 5 months showed prominent global hypoplasia of the cerebellum and pons, generalized mild cerebral atrophy, delayed myelination, and thin dysplastic corpus callosum (Fig. 1A). Extensive metabolic work-up was unremarkable. Electroencephalography (EEG) demonstrated multifocal epileptiform activity (Fig. 1C). On further follow-up he had progressive severe microcephaly, for example aged 23 months HC 40.2 cm (−5.92 *SD*); aged 11 years 44 cm (−6.92 *SD*), and at last follow-up, aged 13 years old HC 47 cm (−4.67 *SD*) and failure to gain weight (18 kg) due to feeding problems. He is non-ambulant, with spasticity, contractures, and reduced muscle mass. Except for smiling, he has not attained any developmental milestones.

His younger brother was born at 40 weeks with a birth weight of 2.5 kg, HC of 32 cm (−1.71 *SD*), an Apgar score of 8 at 1 min and 9 at 5 min. He was also noted to have bilateral cataracts in his postnatal exam. Progressive microcephaly was noted at 2 months of age; at 4 years HC was 43.7 cm (−4.41 *SD*). At last follow-up, he was 6.5 years-old with a weight of 16 kg, and HC 45 cm (−4.89 *SD*). His phenotype was similar to that of his brother, with the exception of epilepsy. It is noteworthy that his MRI demonstrated basal ganglia involvement in addition to PCH (Fig. 1B).

### Genetic workup

Following the WES filtering process, 16 candidate variants were identified, in genes which were either not previously described in association with human disease or were associated with a phenotype not consistent with the patient’s clinical picture. Of these, stood out a homozygous *MED29* missense variant Chr19:398884270T > C (NM_017592); c.416T > C; p.(Leu139Pro), (also referred to as (NM_017592.4); c.353T > C; p.(L118P)) in the proband, which is extremely rare in general population databases (gnomAD frequency = 0), evolutionarily conserved and predicted to be deleterious (Revel 0.9, AlphaMissense 1, BayesDel 0.36). Sanger sequencing confirmed the *MED29* variant in the proband and his affected brother. Familial segregation of the *MED29* variant was consistent with the phenotype (Fig. 1D, E). WES performed on the proband’s affected brother, demonstrated that only 6 of the 16 candidate variants that were identified in the proband, were also identified in a recessive mode in his brother, including homozygous variants in NUCB1 and ZNF575 which are rare in population databases and have a Revel score of >0.8 (Supplementary Table 1). However, none of these 6 candidate variants other than *MED29* appeared relevant. The *MED29* variant was absent from 200 local control samples of healthy individuals with similar ancestry.

### Zebrafish modeling

**Fig. 2:**
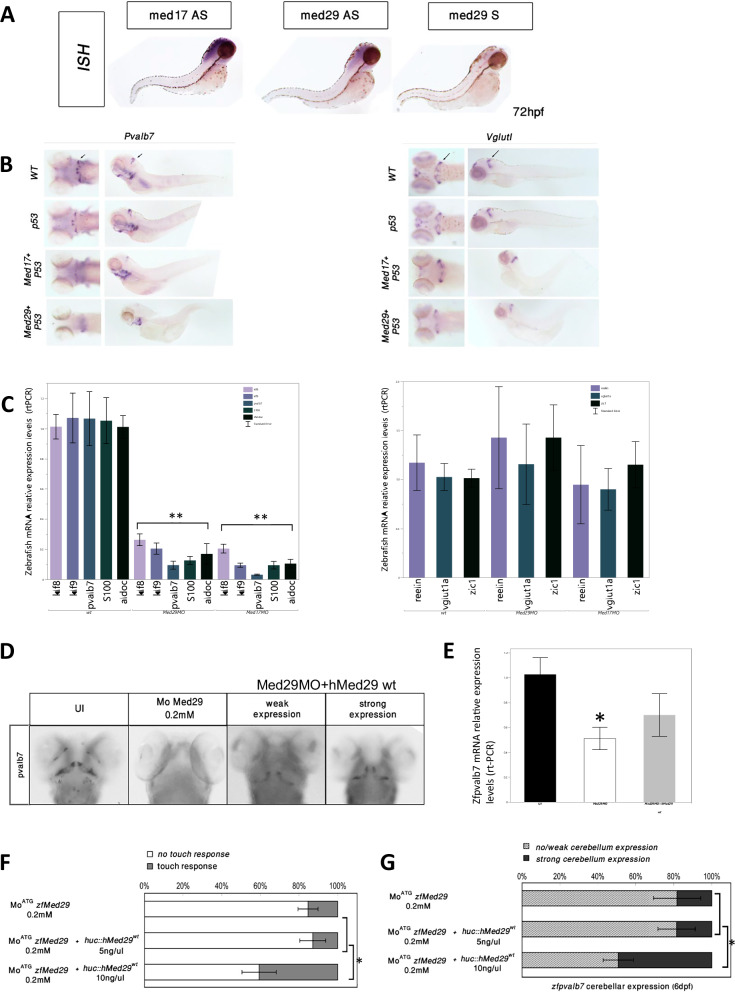
Zebrafish knockdown modeling and rescue. **A** In situ hybridization using antibodies for MED17 and MED29 in a zebrafish embryo model, demonstrating restricted brain expression pattern for both. AS – antisense, S – sense. **B** Immunohistochemistry staining of the zebrafish *MED17*, *MED29*, and *P53* morphants using PValb7 antibodies directed at the GABAergic Purkinje cerebellar cells and Vglut1a antibodies directed at the Glutamatergic Granule cerebellar cells. Left panels – coronal sections, right panels – sagittal sections. WT – wild type. The arrows signify the location of the midbrain-hindbrain boundary (MHB) from which the cerebellar structures develop. *MED17* and *MED29* Morphants exhibit prominent reduced staining of GABAergic Purkinje neurons, but a much lesser reduction in staining of glutamatergic granule neurons. **C** Quantitative analysis of the relative expression GABAergic Purkinje cerebellar cells (on the left) and Glutamatergic Granule cerebellar cells (on the right) in the wt, and *MED29* and *MED17* morphants, by measuring mRNA levels of prototypical genes by rtPCR. A significant reduction of the expression of genes typical to Purkinje cells is seen in both *MED29* and *MED17* morphants, however, there is no reduction in the expression of genes typical to Granules cells. **D** Examples of immunohistochemistry with PValb7 staining, demonstrating normal cerebellar expression in the un-injected (UI) control larvae (left image), absent cerebellar expression in the *MED29* morphant (second from left), and varying degrees of increasing cerebellar expression designated as weak and strong (two panels on the right) following rescue with a plasmid containing human wt *MED29*. **E** Quantitative analysis of the relative expression of pvalb7 gene typical for GABAergic Purkinje cerebellar cells (measured by rtPCR), show normal levels in the wt control larva (left), significantly decreased levels in the *MED29* morphant (middle), and an increase in expression in the *MED29* morphant following rescue with a human wt *MED29*, to levels that are not significantly different from the control wt. **F** Statistical analysis of the proportion of larvae demonstrating a positive touch response in the *MED29* morphants (top bar) and *MED29* morphants following rescue with plasmids containing human wt *MED29* in low concentrations of 5 ng/μl (middle bar) and higher concentrations of 10 ng/μl (bottom bar), showing significantly increased fraction of larva demonstrating positive touch response following rescue with a high concentration but not a low concentration. **G** Statistical analysis of the proportion of larvae demonstrating a “strong” cerebellar expression (indicated by pvalb7 staining as shown in **D** among the *MED29* morphants (top bar) and *MED29* morphants following rescue with plasmids containing human wt *MED29* in low concentrations of 5 ng/μl (middle bar) and higher concentrations of 10 ng/μl (bottom bar), showing significantly increased fraction of larva with strong cerebellar expression following rescue with a high concentration but not a low concentration.

ISH demonstrated strong expression of both *MED17* (used as a known PCH gene for reference) and *MED29* restricted to the central nervous system analog of the zebrafish (Fig. 2A). In both *MED17* and *MED29* morpholino-knockdown (Mok) larvae, a marked reduction of Pvalb^+^ GABAergic Purkinje cerebellar neurons was noted, and to a much lesser extent Vglut^+^ glutamatergic granule cerebellar neurons. There was no reduction Pvalb or Vglut immunohistochemical staining in the control P53 Mok (Fig. 2B). Accordingly, significant (*p* < 0.001) reductions were observed in the mRNA levels of Klf8, Klf9, Pvalb7, S100 and Aldoc genes expressed in GABAergic Purkinje cerebellar neurons, while no significant changes were found in the mRNA levels of Reelin, Vglut1a and zic1 genes expressed in glutamatergic granule cerebellar neurons (Fig. 2C). Touch response was markedly decreased in *MED29* Mok compared to the wt larvae (Supplementary Videos 1 and 2, respectively). Following the rescue with human *MED29* (hMED29), an increase in Pvalb mRNA and immunohistochemical staining was observed (Fig. 2D, E). Significant improvement in locomotor function was observed in *MED29* Mok embryo co-injected with the hMED29 construct (Fig. 2F). A corresponding increase in cerebellar Pvalb7 expression (Fig. 2G) was observed following rescue with the 10 ng/µL concentration constructs, but not with the 5 ng/µL concentration constructs, suggesting a threshold dose-dependent rescue.

### Mice modeling

**Fig. 3:**
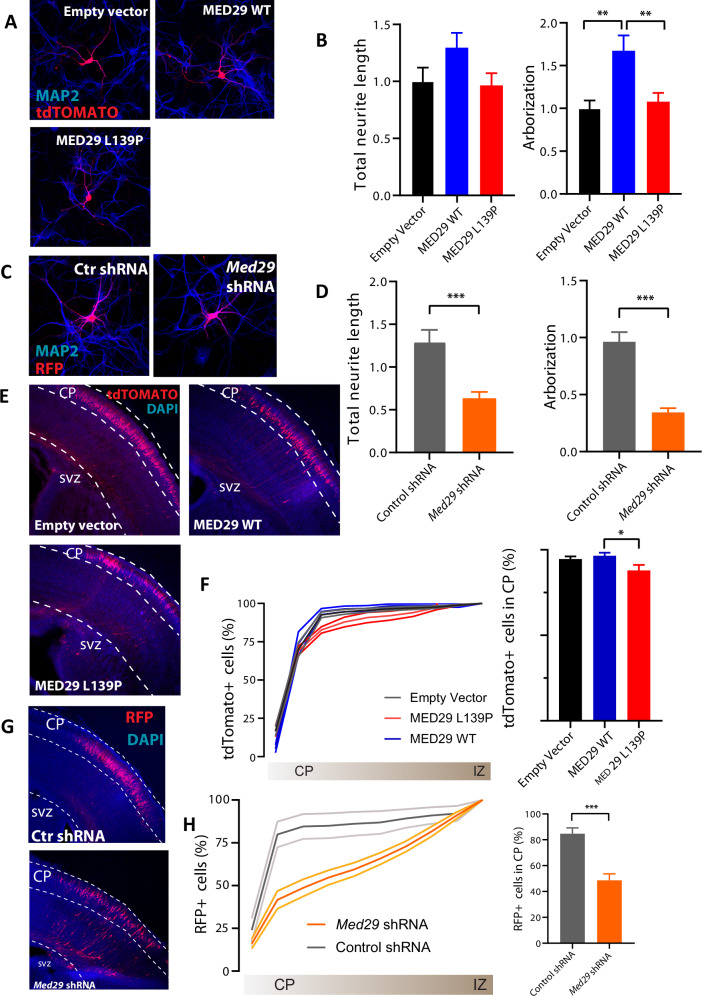
Increased and decreased expression levels of MED29 affect neuronal development in culture; reduced expression levels of MED29 affects neuronal migration in vivo. **A** Representative confocal images of mouse primary hippocampal neurons transfected with empty vector control (left panel), MED29^WT^ (middle panel) or MED29^Leu139Pro^ (right panel). **B** Analysis of the total neurite length (left panel) and arborization (right panel) normalized to control. Overexpression of MED29WT resulted in a trend towards increased neurite length, and significant increase in arborization, compared to empty vector. Overexpression of MED29Leu139Pro showed similar neurite length and arborization as the empty vector control, and was significantly different compared to MED29WT in the number of branches. **C** Representative confocal images of mouse primary hippocampal neurons transfected with control shRNA (left panel) or *Med29* shRNA (right panel). **D** Analysis of the total neurite length (left panel) and arborization (right panel) following *MED29* shRNA normalized to control shRNA. Knockdown of Med29 impaired neuronal development, resulting in significantly shorter dendrites and reduced arborization, compared to control shRNA. Data are presented as mean ± SEM. The number of neurons and number of batches used for analysis for each condition are indicated in the methods section. **p < 0.01. ***p < 0.001. **E** Representative images from mouse brains at postnatal day 1, showing the transfected cells (tdTomato+) from the subventricular zone (SVZ) to the cortical plate (CP) for the empty vector control (left panel), MED29^WT^ (middle panel), and MED29^Leu139Pro^ (right panel). **F** Left, cumulative distribution of the transfected neurons at P1 from the cortical plate (CP) to the intermediate zone (IZ). Right, percentage of neurons reaching the superficial layers of the cortex, showing a small but significant reduction in migration following electroporation with the MED29^Leu139Pro^ vector. **G** Representative images from mouse brains at postnatal day 1 electroporated with control shRNA or *Med29* shRNA, showing the transfected cells (mRFP+) from the SVZ to the cortical plate (CP). **H** Left, cumulative distribution of the transfected neurons at P1 from the cortical plate (CP) to the intermediate zone (IZ). Right, percentage of neurons reaching the superficial layers of the cortex, demonstrating a significant reduction in migration following electroporation with Med29ShRNA. Data are presented as mean ± SEM. Number of images analyzed for each condition is indicated in the methods section. *p < 0.05, ***p < 0.001.

To assess the pathogenicity of the p.(Leu139Pro) missense variant, mouse primary hippocampal neurons were transfected at DIV3 with either empty vector control, *MED29*^*WT*^, or *MED29*^*Leu139Pro*^. After 5 days, neurons were fixed and neuronal morphology was analyzed. Overexpression of *MED29*^*WT*^ resulted in a non-significant increase in neurite length and a significant increase in arborization, compared to empty vector (Neurite length: One-way ANOVA, *F*[2,53] = 2.23, *p* = 0.12; arborization: One-way ANOVA, *F* [2,53] = 8.27, *p* = 0.0007; MED29^WT^ versus empty vector control, *p* = 0.0012, Tukey’s multiple comparisons test; Fig. 3A, B). Overexpression of MED29^Leu139Pro^ did not affect neurite length or arborization compared to the empty vector control, and was significantly different compared to MED29^WT^ in the number of branches (arborization: MED29^Leu139Pro^ versus empty vector control, *p* = 0.89; MED29^Leu139Pro^ versus MED29^WT^, *p* = 0.0048, Tukey’s multiple comparisons test; Fig. 3A, B). These results suggest that MED29^Leu139Pro^ is a loss-of-function variant, as the functional impact of MED29^WT^ overexpression is absent with MED29^Leu139Pro^ overexpression, as is with overexpression of empty vector control. To assess whether loss of MED29 would indeed be damaging for neurons, shRNAs against *Med29* were used to knockdown the gene in primary hippocampal neurons. Morphological analysis revealed that knockdown of *Med29* markedly impaired neuronal development, resulting in significantly shorter dendrites and reduced arborization, compared to control shRNA (neurite length: control shRNA versus *Med29* shRNA, *p* = 0.0051; arborization: control shRNA versus *Med29* shRNA, *p* = 0.0008, unpaired two-tailed Student’s *t*-test; Fig. 3C, D). The knockdown results differ from those of the overexpression of the MED29^Leu139Pro^ or the empty vector, as the endogenous MED29 is still expressed in the overexpression studies, where in the knockdown experiments, the total expression levels of the endogenous protein are reduced.

To further substantiate the role of MED29 in neuronal development, we made use of the *in-utero* electroporation technique [20], inducing overexpression or knockdown of *MED29* in vivo during embryonic neurodevelopment. *In utero* electroporation was performed at E14.5, targeting the somatosensory cortex. At this time point during embryonic neurodevelopment, neurons forming layer 2/3 of the somatosensory cortex are born in the sub-ventricular zone, and subsequently migrate to the CP [21]. Overexpression of MED29^WT^ did not affect neuronal migration of electroporated neurons, as the percentage of electroporated neurons reaching the CP was similar compared to the empty vector control (MED29^WT^ 96%, empty vector control 94%; One-way ANOVA, *F* [2,28] = 3,77, *p* = 0.035; MED29^WT^ versus empty vector control, *p* = 0.8, Tukey’s multiple comparisons test; Fig. 3E, F). Overexpression of the MED29^Leu139Pro^ variant resulted in a small significant difference in migration compared to MED29^WT^, with 87% of the electroporated neurons reaching the cortical plate, but was not significantly different from the empty vector (MED29^Leu139Pro^ versus MED29^WT^, *p* = 0.038; MED29^Leu139Pro^ versus empty vector control, *p* = 0.084, Tukey’s multiple comparisons test; Fig. 3E, F). In contrast, knockdown of *Med29* resulted in a severe migration deficit compared to control shRNA (control shRNA versus *Med29* shRNA, *p* < 0,0001, unpaired two-tailed Student’s *t*-test; Fig. 3G, H), suggesting that MED29 functions critically in neurodevelopment.

## Discussion

We report a novel homozygous *MED29* variant causing a neurodegenerative disorder with PCH. The MED29 subunit (previously known as Intersex) is a highly conserved protein across species, and has been variably reported to be located in the head and the tail of the Mediator complex with recent modeling suggesting that it is located in the upper tail module [22, 23]. To our knowledge, this is the first report implicating recessive germline variants of *MED29* in human disease, although there is evidence of its role as an oncogene in malignancies mediated by transfer-RNA, and that it is overexpressed in pancreatic cancer [24].

The Mediator complex serves as a bridge, connecting the transcription factor with the Pol II machinery, alongside formation of the pre-initiation complex. It also acts a “hub” for coordinating multiple steps in the transcription process, including mRNA and non-coding RNA processing and epigenetic regulation [9]. Increasing evidence has associated other Mediator family members with neurodevelopmental disorders (Table 1). *MED17* was first identified in families of Caucasus Jewish origin presenting in early infancy with spasticity, profound GDD, progressive microcephaly, and epilepsy with early death, though notably without cataracts. MRI in these patients demonstrated marked cerebral, cerebellar, and pontine atrophy and a myelination defect [3, 25]. A milder phenotype with similar signs has also been described in children with *MED17* compound heterozygous variants [6]. Siblings homozygous for *MED20* pathogenic variants were shown to exhibit severe spasticity, dystonia, progressive basal ganglia and cerebellar atrophy, with childhood onset of cataracts [5]. Disease-causing variants in *MED27* have been identified in 57 individuals from 30 families, with GDD ranging from mild to profound, with near universal axial hypotonia and appendicular spasticity, with almost 90% having bilateral cataracts [4, 26]. Microcephaly, epilepsy, dystonia, and spasticity were variable, and MRI demonstrated cerebellar hypoplasia in all patients, with white matter atrophy in most and pontine hypoplasia and basal ganglia atrophy in nearly half. A very severe phenotype including profound GDD, microcephaly, myoclonic seizures, a minority with cataract, and premature death, was recently described in 7 individuals with *MED11* defects [2]. Brain MRI in these patients showed progressive cerebral and cerebellar atrophy, cerebral dysgyria, and variable basal ganglia degeneration, but without clear signs of pontine involvement. Of note, MED17 and MED11 subunits are thought to be in the head module of the Mediator complex, while MED27 is situated in the upper tail module with interactions with the head module [26]. Interestingly, in silico modeling has demonstrated that an N-terminal region of MED27 forms a heterodimeric helical bundle with MED29 [26] (https://michelanglo.sgc.ox.ac.uk/r/med27). They reflect the severe end of the spectrum of MEDopathies characterized by severe phenotypes with most notable cerebral and cerebellar atrophy, associated with microcephaly and severe-profound GDD.

**Table 1:** Clinical phenotype of previously described MEDopathies and individuals described here.

	MED12 [29–31]	MED17 [3, 6, 25]	MED20 [5]	MED25 [8]	MED27 [4, 26]	MED11 [2]	MED29 current work
Cerebellar hypoplasia/atrophy	+/-	++	++	-	++	+	++
Cerebral atrophy	+/-	++	++	+	+	++	++
Basal ganglia abnormalities	-	-	++	-	+	+	+
Myelination abnormality	-	++	-	n/a	+	+	++
Corpus callosum abnormality	+	++	++	-	+	-	++
Microcephaly	+/-	++	++	++	+	++	++
Spasticity	-	++	++	n/a	+	+/-	++
Axial hypotonia	+	++	++	++	+	++	++
Dystonia	-	-	+	+	+	+	-
Ataxia	+/-	+/-	-	n/a	+	n/a	n/a
Behavioral abnormalities	++	-	-	n/a	+/-	n/a	n/a
GDD/ID	+	++	++	++	++	++	++
Seizures	+/-	++	-	+/-	+	+	+
Cataracts	-	-	+	+	+	+/-	++
Dysmorphism	++	-	-	++	+	++	-
Hearing loss	+/-	-	-	-	+/-	++	n/a
Cardiac defects	-	-	-	+	-	+/-	n/a
Mode of inheritance	X-linked	AR	AR	AR	AR	AR	AR

++ Always observed, + Frequently observed, +/- Variably observed, - Never observed. n/a – no available data.

However, there is increasing data of milder phenotypes lacking the striking neuroradiological signs described above, in disorders associated with other subunits. Among these are *MED12* (OMIM 309520), which is related to X-linked inherited intellectual disability and neuropsychiatric disorders, as well as *MED13* (OMIM 618009) and *CDK8* (OMIM 618748) variants in patients with facial dysmorphism and mild hypotonia, ID, and behavioral disturbances [27–32]. Patients with *MED25* biallelic variants have microcephaly, congenital cataract, and severe developmental delay, yet lack cerebellar and pontine changes [8].

In common with other patients reported with variants in *MED11*, *MED17*, *MED20*, and *MED27*, we demonstrate that *MED29* biallelic variants cause progressive microcephaly, intellectual disability, spasticity, and cerebral atrophy, in addition to the striking pontocerebellar hypoplasia with likely progressive basal ganglia involvement. Of note, while the distinction between PCH and cerebellar atrophy is often unclear and can require serial neuroimaging to establish, we describe this entity as *MED29*-associated PCH with the impression that developmental hypoplasia is the prominent phenotype, as shown in the proband’s MRI aged 5 months. However, some degree of progressive cerebellar atrophy also exists, as evident in his brother’s imaging at a later age and from the progressive microcephaly. Our proband had epilepsy but not his younger brother, and therefore it is unclear whether the epilepsy can appear later through childhood or is an inconsistent feature of *MED29*-associated PCH. Congenital cataracts were present in both siblings, a common yet not universal feature in the other Mediator-associated neurodevelopmental disorders, suggesting that the MED complex functions as a unit with some subunits having particularly similar roles.

To model the different clinical aspects of MED29 dysfunction we used three different modalities—zebrafish morphants, mouse primary hippocampal cultures, and mouse embryos. Consistent with zebrafish models of TSEN54-related PCH [33], we also demonstrated structural abnormalities correlating with PCH in our zebrafish model of *MED29* morphants as well as in *MED17* morphants, which we used as a MED-related PCH prototype. Interestingly, both *MED17* and *MED29* morphants exhibited reduced expression of GABAergic Purkinje neurons, but with a milder effect of glutamatergic granule neurons, in line with the findings in the PRDM13 model, causing a similar PCH phenotype [34], suggesting shared disease processes with Mediator diseases impairing Purkinje cells differentiation specifically. *MED29* morphants also exhibited markedly reduced touch responses correlating with the severe motor dysfunction of the patients. Both structural and functional abnormalities were restored by rescue with expression of human wild-type *MED29* in a dose-dependent manner, further supporting the genotype-phenotype association. While MED29wt overexpression resulted in increased neurite length and arborization, overexpression of both empty vector and MED29^Leu139Pro^ variant did not affect neuronal morphology, thereby supporting a loss-of-function mechanism of the Leu139Pro variant and further validating the results in the knockdown models. ShRNA-mediated knockdown of *MED29* in mouse hippocampal cultures significantly reduced neurite length and dendritic arborization, likely consistent with the severe microcephaly of the patients. Taken together, these results suggest that the expression level of MED29 needs to be tightly regulated, as either too high or too low expression levels both result in altered neuronal morphology. Electroporation studies in mice embryos demonstrated a migration defect in both overexpression of the MED^Leu139Pro^ variant and by ShRNA-mediated knockdown of MED29, suggesting that MED29 has a role in early development of the brain.

In conclusion, we demonstrate that *MED29* is a novel disease-causing gene of a severe neurodegenerative disease with a distinct phenotype including PCH and cataract. The majority of PCH-related genes involve RNA splicing and charging. Given the common clinical and radiological features of Mediator neurodegenerative diseases and consistent zebrafish functional models, including our findings together with the known role of the Mediator complex in Poll II transcription [35], we propose that they should be included in the evolving PCH classification [36]. Further studies should explore the prevalence of the *MED29* variant described here in the Middle Eastern Arab population to assess for the presence of a founder effect. Additional functional work is warranted to characterize downstream effects of MED29 defects on RNA transcription.

## Supplementary information

Supplementary video 1

Supplementary video 2

Supplementary Table 1

Supplementary video Legends

## Data Availability

The data generated in this study can be found within the article. Raw data is available from the corresponding author request.
