# Supplementary material for: Biallelic MED29 variants cause pontocerebellar hypoplasia with cataracts
Source: Eur J Hum Genet. 2025 Jul 31;33(10):1271–80. doi: 10.1038/s41431-025-01918-6 (PMC12480692; doi:10.1038/s41431-025-01918-6)
Supplement: Supplementary file 3 — Supplementary Table 1 [file 41431_2025_1918_MOESM3_ESM.docx]

|  | **Gene name** | **Genomic location** | **Function** | **Zygosity in Proband** | **Zygosity in affected brother** |
| --- | --- | --- | --- | --- | --- |
| 1 | *KIF20B* | Chr10:91533776C>T | Stop Gain | Homozygous | Heterozygous |
| 2 | *MED29* | Chr19:39884270T>C | Missense | Homozygous | Homozygous |
| 3 | *NUCB1* | Chr19:49416753A>C | Missense | Homozygous | Homozygous |
| 4 | *USP14* | Chr18:211127A>G | Splice Site | Homozygous | Heterozygous |
| 5 | *MAU2* | Chr19:19459754C>T | Splice Site | Homozygous | Heterozygous |
| 6 | *ZNF575* | Chr19:44039472G>A | Missense | Homozygous | Homozygous |
| 7 | *SOD2* | Chr6:160114261C>G | Splice Site | Homozygous | - |
| 8 | *WIPI1* | Chr17:66417651A>G | Splice Site | Homozygous | - |
| 9 | *KIF11* | Chr10:94366137A>G | Missense | Homozygous | Heterozygous |
| 10 | *SMC6* | Chr2:17912340A>G | Splice Site | Homozygous | - |
| 11 | *RWDD4* | Chr4:184580361A>C | Splice Site | Homozygous | - |
| 12 | *PLAUR* | Chr19:44156378G>A | Splice Site | Homozygous | Homozygous |
| 13 | *LDB3* | Chr10:88447027T>C | Splice Site | Homozygous | - |
| 14 | *BEND2* | ChrX:18213485G>A | Missense | Hemizygous | Hemizygous |
| 15 | *DIAPH2* | ChrX:96013203C>G | Missense | Hemizygous | Hemizygous |
| 16 | *IKZF2* | Chr2:213921714G>A  Chr2:214012403_214012403delAA | Splice Site  Frame Shift | Compound heterozygous | -  - |

Supplementary Table 1: Results of the whole exome sequencing filtering process in the proband and his brother.

16 candidate variants survived the filtering process of the WES in the proband. Of these, only 6 including the *MED29* variant were later on found in a recessive mode in his affected brother. "–" signifies both alleles were wild type.
