# Supplementary material for: Biallelic MED29 variants cause pontocerebellar hypoplasia with cataracts
Source: Eur J Hum Genet. 2025 Jul 31;33(10):1271–80. doi: 10.1038/s41431-025-01918-6 (PMC12480692; doi:10.1038/s41431-025-01918-6)
Supplement: Supplementary file 4 — Supplementary video Legends [file 41431_2025_1918_MOESM4_ESM.docx]

**Video 1****.** **Intact touch response of wt zebrafish.** Representative video of wt larvae at 6 dpf, taken under a dissecting stereoscope with a DP72 digital camera (Olympus), displaying normal bursting locomotion in response to light contact with a fine hairbrush.

**Video 2.** **Impaired touch response of *MED29* morpholino-knockdown zebrafish.** Representative video of *MED29* morphant larvae at 6 dpf, taken under a dissecting stereoscope with a DP72 digital camera (Olympus), demonstrating reduced acceleration during bursting locomotion in response to light contact with a fine hairbrush.
